# Fatigue Symptoms Influence Effort-Based Decision-Making in Major Depressive Disorder

**DOI:** 10.1016/j.bpsgos.2026.100780

**Published:** 2026-06-24

**Authors:** Grace E. Steward, Adam J. Culbreth, Fernando S. Goes, Vikram S. Chib

**Affiliations:** a Department of Biomedical Engineering, Johns Hopkins University School of Medicine, Baltimore, Maryland; b Maryland Psychiatric Research Center, Department of Psychiatry, University of Maryland School of Medicine, Baltimore, Maryland; c Department of Psychiatry and Behavioral Sciences, Johns Hopkins University School of Medicine, Baltimore, Maryland; d Kavli Neuroscience Discovery Institute, Johns Hopkins University, Baltimore, Maryland; e Kennedy Krieger Institute, Baltimore, Maryland

**Keywords:** Behavior, Decision making, Effort, Fatigue, Major depressive disorder

## Abstract

**Background:**

Fatigue is a central symptom of major depressive disorder (MDD). Studies in healthy individuals suggest that fatigue reduces willingness to exert effort, but its role in effort-based decision making in MDD remains unclear. Here, we examined how fatigue and depressed mood influence cognitive effort–based choices in individuals with MDD.

**Methods:**

Healthy participants (*n* = 26) and individuals with MDD (*n* = 18) completed a forced-choice paradigm in which they chose between a low-effort cognitive task for a small reward and a more demanding task for a larger reward. Participants also completed the Beck Depression Inventory (BDI) and Modified Fatigue Impact Scale (MFIS) to assess depressive symptoms and fatigue severity.

**Results:**

Individuals with MDD reported significantly greater fatigue severity compared with healthy control participants. Across participants, higher MFIS scores were associated with a reduced willingness to select cognitively effortful options, particularly at higher effort levels. Fatigue severity explained choice behavior better than diagnostic group or depressive symptom severity measured by the BDI. These findings suggest that fatigue, rather than depressed mood alone, is a key factor shaping cognitive effort–based decision making in MDD.

**Conclusions:**

Our findings demonstrate that fatigue substantially influences willingness to exert cognitive effort. These results provide insight into mechanisms underlying reduced goal-directed behavior in depression and may help guide interventions targeting fatigue-related motivational impairments.

## Plain Language Summary

People with major depressive disorder (MDD) often experience fatigue and difficulty engaging in mentally demanding activities, but it remains unclear how fatigue influences decisions about cognitive effort. In this study, individuals with MDD and healthy control participants completed a task in which they chose whether to engage in different levels of cognitive effort for monetary rewards and completed questionnaires measuring fatigue and depressive symptoms. We found that individuals with greater fatigue symptoms were less willing to choose more cognitively demanding options as effort requirements increased. These findings suggest that fatigue may play an important role in motivational difficulties in depression and may help explain why some individuals with MDD avoid cognitively effortful activities.

Major depressive disorder (MDD) profoundly affects various aspects of individuals’ lives, including motivation, decision making, and willingness to exert effort. Diagnostic criteria for MDD include fatigue as a core symptom (1), which can affect these behaviors, disrupting the ability to engage in tasks that require cognitive effort. Although there has been a great deal of work examining how MDD influences decisions to exert effort, and fatigue is a well-established symptom, there is limited understanding of how MDD-induced fatigue affects decision making and motivated performance. In this study, we examine how individuals with MDD trade cognitive effort for rewards and how fatigue and depressive symptoms shape this decision-making process.

Effort-based decision-making has been proposed as a behavioral approach for understanding motivation. In this framework, the willingness to exert effort is influenced by the subjective value of the rewards associated with a particular outcome, as well as the trade-off between the reward and the effort required to obtain it. Effort-based decision-making studies have been applied in the context of physical (2, 3, 4, 5, 6, 7, 8, 9, 10) and cognitive (8,11, 12, 13) effort in healthy individuals, revealing that the willingness to undertake effort increases as the levels of prospective effort-contingent reward rise, and that the subjective value of effort increases through repeated fatigue-induced exertion.

Recently, several studies have explored effort-based decision-making in individuals with MDD, focusing on both cognitive and physical effort. Although many studies have reported that individuals with MDD are less willing to exert cognitive (14, 15, 16, 17, 18) and physical (17, 18, 19, 20, 21, 22, 23, 24) effort compared with healthy individuals, findings across studies have been somewhat inconsistent, with some reporting reductions in effort expenditure in individuals with MDD, whereas others have observed relatively preserved effort-based choice behavior or effects that vary across symptom dimensions and diagnostic categories (19,25, 26, 27). One potential explanation for these inconsistencies is that prior work has largely focused on diagnostic group differences without explicitly considering variability in fatigue-related symptoms, even though fatigue is a hallmark feature of MDD that may substantially influence willingness to engage in effortful behavior. Consistent with this idea, studies of effort-based decision-making have shown that feelings of fatigue significantly influence choice behavior (2,4,6,7,11,28,29), and individuals with depression tend to perceive activities as more effortful than healthy controls (16,30). However, there has been limited investigation into how fatigue symptoms specifically contribute to effort-based decision-making in individuals with MDD. Therefore, this study was designed to examine whether fatigue-related symptoms contribute to variability in cognitive effort-based decision-making in MDD.

In this study, we investigate differences in cognitive effort-based decision-making between individuals with MDD and healthy control participants and evaluate whether fatigue or depressed mood provides a better explanation for these behavioral differences. We hypothesize that individuals with MDD would be less willing to exert the same cognitive effort for monetary rewards than healthy control participants. Additionally, we expect that fatigue symptoms in MDD will significantly influence these decision-making differences and offer greater insight into effort-based decision-making than depression symptoms alone. These hypotheses are built on prior research examining decision-making in individuals with MDD, along with studies of fatigue in healthy individuals, to provide an account of how fatigue might influence choice in individuals with depression.

## Methods and Materials

### Participants

This study was approved by the Johns Hopkins School of Medicine Institutional Review Board. Participants for this study were recruited from the Johns Hopkins University community via online postings. We collected data from 21 participants meeting DSM-IV criteria for MDD (with no history of psychosis) and 29 healthy control participants from the same community with no self-reported history of neurologic or psychiatric disorders. Participants were excluded if they met the criteria for substance dependence or abuse evaluated under the Mini-International Neuropsychiatric Interview (31). Medication history was collected from the MDD cohort (Table S1). No participants in the control cohort reported having ever taken a psychotropic medication. Informed consent was obtained before commencement of the study.

After collection, participant data were excluded if their choices indicated aversion to reward during the experiment. This was determined using a logistic regression of choice data. Participants with a negative weight for reward were excluded. Three participants were removed from each group, leaving a total of 18 participants with MDD and 26 healthy control participants.

There were no significant differences in age, sex, race, ethnicity, or income between cohorts (Table 1). However, a marginal difference in years of education was found between the MDD group and the control participants group (*t*_42_ = −1.867, *p* = .069, with those in the MDD group having nominally fewer years of education).

**Table 1:** Demographic Summary of the Study Participants

Demographic Characteristics	Group		Statistical Test	
	Healthy Control Participants	Participants With MDD	Test Statistic	*p* Value
Age, Years	24.46 (4.02)	22.95 (3.98)	*t*_42_ = −1.236	.226
Gender, Female	72.2%	69.2%	χ^2^_1_ = 0.046	.831
Years of Education	17.26 (3.24)	15.67 (1.97)	*t*_42_ = −1.867	.069
Racial Identity				
Asian	61.5%	38.9%	χ^2^_3_ = 2.187	.198
Other	7.7%	11.1%	χ^2^_3_ = 0.150	.698
White	30.8%	50.0%	χ^2^_3_ = 1.659	.139
Symptom Scales				
BDI score	3.08 (2.97)	25.11 (11.58)	*t*_42_ = −9.319	8.832 × 10^−12^

Values are presented as mean (SD) or %, unless otherwise specified.

BDI, Beck Depression Inventory; MDD, major depressive disorder.

#### Diagnostic and Symptom Assessment

Diagnoses in MDD cases were performed using the Diagnostic Interview for Genetics Studies (DIGS) version 4.0 (32) based on DSM-IV criteria. DIGS assessments were not performed on healthy control participants. To assess symptoms of depression and fatigue, all participants completed the Beck Depression Inventory (BDI) (33) and the Modified Fatigue Impact Scale (MFIS) (34).

### Cognitive Effort Task

**Figure 1:**
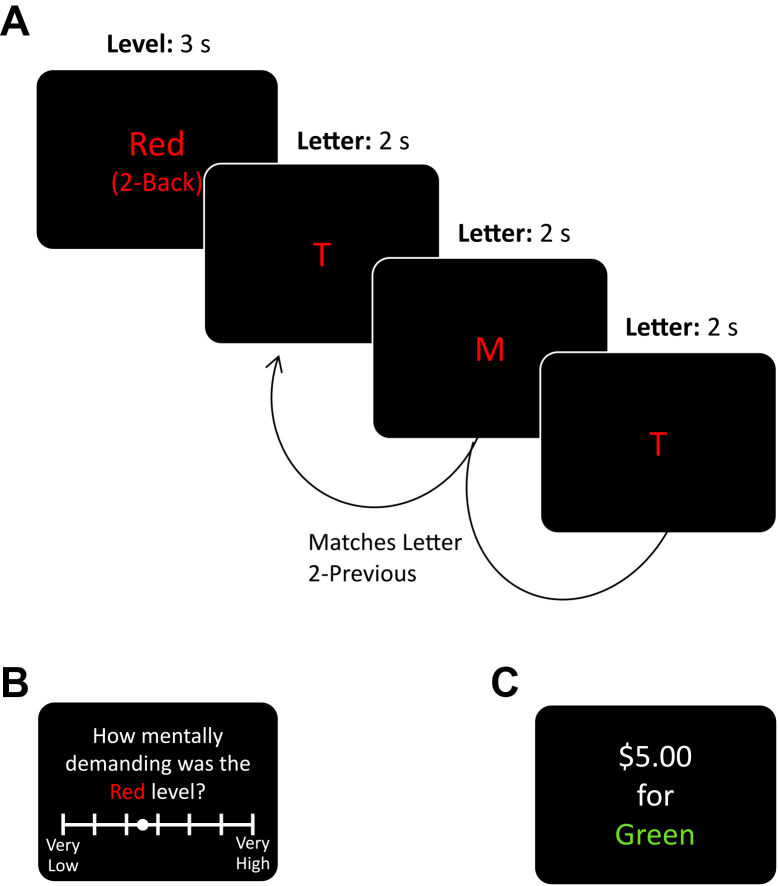
Experimental design. **(A)** Participants first associated different effort levels of the n-back task with distinct colors. During the n-back cognitive effort task, participants identified when a letter presented on the screen matched the one presented n letters previously. **(B)** Following this association phase, participants rated the difficulty of each color level on a continuous scale from very low to very high mental demand. **(C)** During choice trials, participants chose between a default effort/reward (1 back for $1) option and an option with varying levels of effort for different reward amounts.

Before the study began, participants were informed that they would receive a $15 show-up fee, regardless of their performance during the task, but that they would also earn additional money based on their choices and performance. Our paradigm design was influenced by a previously designed cognitive effort task from Westbrook *et al.* (12). However, we adjusted the choice phase of the paradigm so that the range of reward and effort was not explicitly linked in a progressive ratio task. In this way, effort levels and rewards were orthogonalized in our experimental paradigm, allowing us to analyze the effects of reward and effort separately (11). We began our experiment by familiarizing participants with the cognitive effort needed to complete a level of the n-back task (Figure 1A). Each n-back task consisted of 40 sequentially presented letters. Within this sequence, there were 10 target letters that a participant had to identify as the same letter n letters prior. Participants had approximately 2 seconds to identify whether the current letter on the screen was one of these targets. Once a participant entered their choice, the next letter would be presented. Participants had to correctly identify 50% of these targets to succeed in the task. Feedback was presented at the end of each sequence. If participants correctly identified 50% of the targets, they received a success message on the screen. If they failed to achieve 50% success, they received a message of please try harder and continued to the next n-back level. To avoid anchoring effects of subsequent difficulty ratings to numeric effort levels, participants associated each of the 4 n-back task levels (n = 1–4) with a distinct color. Participants experienced each effort level 3 times in succession. Each effort level was presented in random order without replacement.

Following this association phase, participants underwent an assessment phase to ensure that they had developed salient associations between levels of cognitive effort and their colors. They rated the mental challenge of each n-back level on a continuous scale from very low to very high (Figure 1B).

Finally, participants underwent a choice phase in which they could opt for a default option to complete a 1-back for $1, or engage in a variable level of cognitive effort for a variable monetary reward (Figure 1C). This phase consisted of 24 unique offers that orthogonally spanned 3 effort levels and monetary rewards ranging from $1 to $8, in increments of $1 (Table S2). Importantly, participants made prospective decisions regarding effort expenditure during this phase and did not repeatedly exert effort while making choices, minimizing the accumulation of task-induced fatigue during decision-making. To ensure this choice paradigm was incentive-compatible, at the end of the experiment, 2 offers from the choice phase were randomly selected and implemented. The participant then executed the chosen option, exerting the associated effort and earning the reward. Participants needed to succeed in the effort task to receive the reward, and they were given 3 opportunities to succeed.

### Data Analysis

#### Task Performance

Task accuracy was calculated as the ratio of targets and nontargets that were correctly identified out of 40 total letters in each n-back trial during the association phase. To evaluate differences in task accuracy between MDD and control groups, we constructed a linear mixed-effects model that related task accuracy to n-back level with group as a categorical fixed effect and participant as a random effect to account for inter-individual differences:

(1) $$A\left(t\right)\;∼\;N\left(t\right)\;+\;\text{MDD}\;+\;N\left(t\right)\;\times \;\text{MDD}\;+\;\left(1\;|\;P\left(t\right)\right)$$

Parameters were estimated using the fitlme function of MATLAB (version 2023a, The MathWorks, Inc.) (35), with relative coding of dummy variables and the maximum likelihood fit method. This model included task accuracy *A*(*t*) and n-back level *N*(*t*) as continuous variables, whereas MDD represents a categorical variable of the diagnostic group (MDD/control), and *P*(*t*) is a categorical variable for each participant for a particular trial *t*.

#### Task Difficulty Ratings

To analyze differences in task difficulty ratings between cohorts, we constructed a linear mixed-effects model:

(2) $$D\left(t\right)\;∼\;N\left(t\right)\;+\;\text{MDD}\;+\;\;N\left(t\right)\;\times \;\;\text{MDD}\;+\;\left(1\;|\;P\left(t\right)\right)$$

This model included task difficulty ratings *D*(*t*) and n-back level *N*(*t*) as continuous variables. Mirroring the task-performance model, MDD represents a categorical variable of the diagnostic group and *P*(*t*) are categorical identifiers for a participant of a given trial *t*. This model was estimated using the same model estimation procedure as the task-performance model.

#### Analysis of Effort-Based Decision-Making Data

To examine how choice behavior varied as a function of effort cost, reward value, and group, we used a hierarchical generalized logistic mixed-effects model to capture all available data from the effort-based choice trials. We assumed that choice would be affected by the effort and reward of the presented option and that there would be an effect of cohort on choice, all of which were modeled as fixed effects. We expected random biases attributable to each participant, modeled as a random intercept:

(3) $$C\left(t\right)\;∼\;E\left(t\right)\;+\;R\left(t\right)\;+\;\text{MDD}+E\left(t\right)\;\times \;\text{MDD}\;+\;R\left(t\right)\;\times \;\;\text{MDD}\;+\;\left(1|P\left(t\right)\right)$$

where *C*(*t*) is the choice being made on a given trial *t* (0 = default option, 1 = presented option), *E*(*t*) is the effort of the presented option encoded as the n in the n-back being offered, *R*(*t*) is the reward corresponding to the successful completion of the effort level, MDD is a categorical variable of group, and *P*(*t*) is a categorical participant identifier. Effort and reward were linearly standardized on the interval of (0,1) to preserve the significance of sign. Models were estimated with the fitglme function of MATLAB (version 2023a, The MathWorks, Inc.) (35) using the Laplace approximation of maximum likelihood (36,37) and an assumption of a binomial distribution of the binary response variable of choice.

It should be noted that the mixed-effects models included participant-specific random intercepts to account for baseline differences in choice behavior. Although random slopes for effort and reward can, in principle, capture inter-individual variability in sensitivity to these predictors, the present analyses focused on estimating population-level effects. Given the number of observations per participant relative to model complexity, a more parsimonious random-effects structure was adopted to avoid overparameterization and ensure stable model estimation (38,39).

To confirm that performance on the n-back tasks was not a primary factor influencing choice behavior, we also compared the main choice model (3) to a model in which n-back level was replaced by participants’ percent success at each n-back level from the association phase *E*_acc_(*t*).

(4) $$C\left(t\right)\;∼\;E_{\text{acc}}\left(t\right)\;+\;R\left(t\right)\;+\;\text{MDD}\;+\;E_{\text{acc}}\;\times \;\text{MDD}\;+\;R\left(t\right)\;\times \;\text{MDD}+\left(1|P\left(t\right)\right)$$

Because individuals with MDD had significantly higher levels of depressive symptoms and fatigue ratings, as measured by the BDI and MFIS scales, and given our relatively small cohort size, we fit another set of logistic mixed effects models to examine how participants’ choices varied as a function of effort and reward and how they interacted with the BDI and MFIS scales and subscales. We fit separate models for each scale and compared Akaike information criterion (AIC) to evaluate how well the different models explained choice. These models had the form.

(5) $$C\left(t\right)\;∼\;E\left(t\right)\;+\;R\left(t\right)\;+\text{Scale}\;+E\left(t\right)\;\times \;\text{Scale}\;+\;R\left(t\right)\;\times \;\text{Scale}+\left(1|P\left(t\right)\right)$$

Here Scale represents the scale used for a given model. Separate choice models were fit using MFIS subscale scores (cognitive, physical, and psychosocial) and BDI subscale scores (cognitive-affective and somatic) in place of the corresponding total questionnaire scores. These analyses were performed to determine whether specific dimensions of fatigue or depressive symptoms better accounted for variability in prospective cognitive effort-based decisions.

## Results

### Differences in Choice Preferences Between Individuals With MDD and Healthy Participants

**Figure 2:**
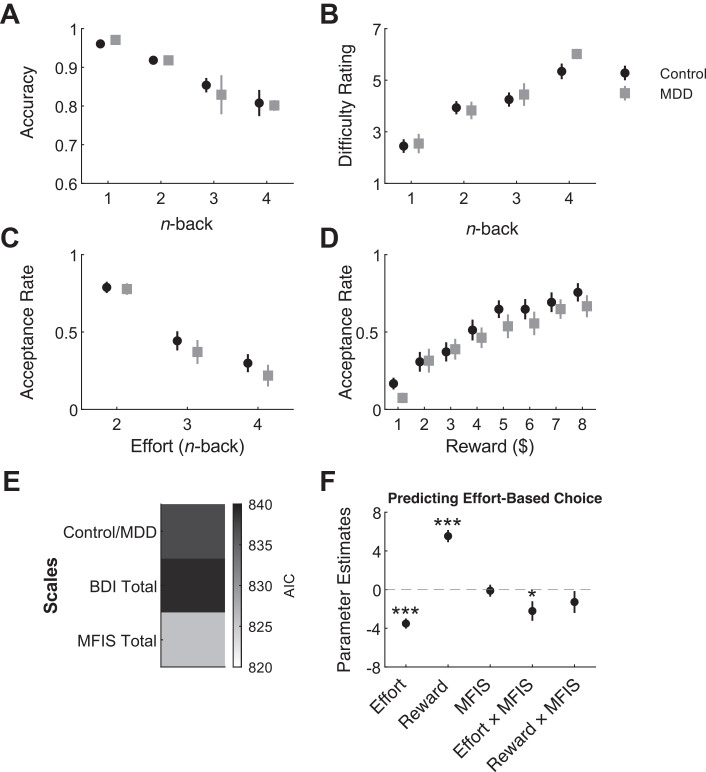
Behavioral data. **(A)** Working memory task accuracy as a function of n-back level for the major depressive disorder (MDD) and control groups. **(B)** Participants’ ratings of working memory task difficulty as a function of the n-back level. Probability of accepting the nondefault effort/reward option as a function of cognitive effort level **(C)** and reward **(D)**. Error bars represent standard error. **(E)** Heat map illustrating Akaike information criterion (AIC) for separate models of effort-based choice that included group (control/MDD), Beck Depression Inventory (BDI), and Modified Fatigue Impact Scale (MFIS) scores as factors. The model that best described the choice data included MFIS score as a factor. **(F)** Parameter estimates from a logistic mixed-effects model predicting choice of the nondefault effort/reward option. Error bars indicate standard error of mean. ∗*p* < .05, ∗∗∗*p* < .001.

To evaluate differences in effort perception or task performance between the MDD and control groups, we analyzed both cognitive exertion performance and effort perception across the n-back task levels. We did not find significant differences in task accuracy (Figure 2A, Table S3) (linear mixed-effects model, *t*_173_ = −0.257, *p* = .798) or in the perceived difficulty of the task (Figure 2B, Table S4) (linear mixed-effects model, *t*_173_ = 0.984, *p* = .327) between groups. These results suggest that performance and the perception of cognitive effort were matched between groups, allowing us to infer differences in choice that are not the byproduct of differences in perception or performance.

Participants in both the MDD and control groups’ likelihood of accepting the nondefault effort/reward option decreased with increasing effort levels (Figure 2C, Table S5) (β_E_ = −4.126, *t*_1037_ = −11.054, *p* = 6.275 × 10^−27^) and increased with increasing reward levels (Figure 2D, Table S5) (β_R_ = 4.870, *t*_1037_ = 10.903, *p* = 2.799 × 10^−26^). However, a model examining the effect of group on the propensity to choose the nondefault option did not find a significant main effect of group (β_MDD_ = −0.399, *t*_1037_ = −0.755, *p* = .450) (Table S5), or interactions between group and reward (β_R×MDD_ = 0.039, *t*_1037_ = 0.054, *p* = .957), or group and effort (β_E×MDD_ = −0.758, *t*_1037_ = −1.207, *p* = .228).

We also performed a control analysis to confirm that choice was driven by n-back effort level and by effort performance. When evaluating the influence of effort performance, we found that n*-*back level was a better predictor of choice than task accuracy (n-back level AIC = 837, task accuracy AIC = 1085). Additionally, task accuracy was not a significant predictor of choice (β_Accuracy_ = −0.555, *t*_1037_ = −1.437, *p* = .151) (Table S6). This suggests that differences in choice behavior between the control participants and MDD participants are more closely related to the n-back level itself than to performance on the cognitive effort task.

### Separating Fatigue and Mood Symptoms

We next evaluated whether depression or fatigue scores across participants moderated the effects of reward and effort on choice. We found that total scores on both the BDI (*t*_42_ = −9.319, *p* = 8.832 × 10^−12^) and MFIS (*t*_42_ = −7.255, *p* = 6.284 × 10^−12^) were significantly higher for individuals with MDD than for control participants, indicating that participants with MDD exhibited higher levels of depression and fatigue. Total BDI and MFIS scores were also significantly correlated (ρ = 0.845, *p* = 1.711 × 10^−13^), suggesting a link between these measures. We found that propensity to accept the nondefault effort/reward option was modulated by the interaction between individuals’ MFIS scores and effort cost (Figure 2E, Table S7) (β_E×MFIS_ = −2.214, *t*_1037_ = −2.190, *p* = .029); however, this relationship was not present in a separate model examining the interaction between effort cost and BDI (β_E×BDI_ = 0.573, *t*_1037_ = 0.561, *p* = .575) (Table S8). The model including MFIS (AIC = 826) described choice better than the model including BDI (AIC = 839). These findings suggest that fatigue symptoms may play an important role in driving effort-based decision-making in MDD.

**Figure 3:**
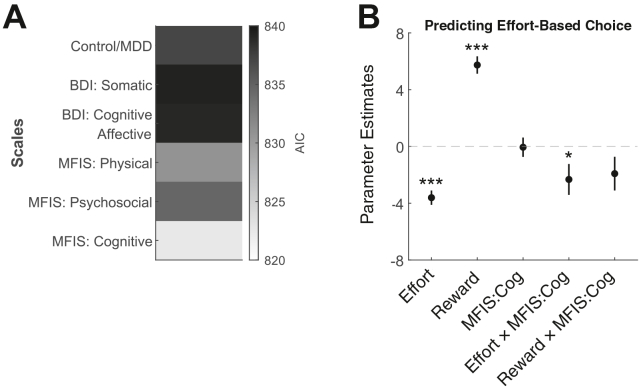
Modified Fatigue Impact Scale (MFIS) and Beck Depression Inventory (BDI) subscales. **(A)** Heat map illustrating Akaike information criterion (AIC) for separate models of effort-based choice that included group (control/major depressive disorder [MDD]), BDI, and MFIS subscales as factors. The model that best described the choice data included the MFIS subscale score for cognitive fatigue as a factor. **(B)** Parameter estimates from a logistic mixed-effects model predicting choice of the nondefault effort/reward option. Error bars indicate standard error of mean. ∗*p* < .05, ∗∗∗*p* < .001.

To further examine how different dimensions of the MFIS and BDI might relate to cognitive effort-based decision-making in MDD, we evaluated how subscales of these scores related to participants’ choices. We examined separate choice models that included the physical, psychosocial, and cognitive subscales of the MFIS and the cognitive and somatic subscales of the BDI. The models that best described the data were those that included the MFIS cognitive subscale (Figure 3A). Again, the Cognitive Subscale of the MFIS modulated individuals’ sensitivity to effort value when making cognitive effort-based decisions (β_E×MFIS:Cog_ = −2.324, *t*_1037_ = −2.136, *p* = .033) (Table S9). These results further support the idea that fatigue is an important factor to consider when evaluating how individuals with MDD make decisions that involve cognitive exertion.

## Discussion

We examined differences in effort-based decision-making between individuals with MDD and healthy control participants and assessed how fatigue and depressive symptoms influence prospective cognitive effort decisions. Individuals with MDD exhibited significantly higher levels of fatigue compared with healthy control participants. Across participants, we observed an interaction between fatigue severity and prospective effort level on choice behavior, such that individuals with higher fatigue scores were progressively less willing to choose cognitively demanding options as effort requirements increased. Choice behavior was better explained by fatigue-related modulation of effort cost than by diagnostic group or depressive symptom severity. Importantly, we did not find evidence that fatigue or depressive symptoms reduced reward sensitivity. Together, these findings highlight fatigue as a critical factor influencing motivation and cognitive effort allocation in depression and suggest that altered sensitivity to effort costs may contribute to motivational impairments observed across psychiatric and neurologic disorders.

Our results are broadly consistent with previous studies implicating altered effort-based decision-making in MDD (15,17,18,23,40,41). However, rather than observing a direct group difference in choice behavior, we found that individuals with MDD reported significantly greater fatigue, and that fatigue severity modulated prospective effort-based decisions across participants. These findings suggest that variability in fatigue symptoms may underlie individual differences in cognitive effort allocation that are not fully captured by diagnostic group alone. Specifically, higher fatigue was associated with greater sensitivity to prospective effort costs, resulting in a reduced willingness to choose cognitively demanding options as effort requirements increased. It is also possible that the relatively modest sample size of the current study limited our ability to detect more subtle between-group differences in choice behavior.

Our paradigm orthogonalized prospective effort and reward, allowing us to isolate their independent effects on decision-making. Importantly, participants made decisions about prospective cognitive effort demands without repeatedly exerting effort during the choice phase of the task. This design minimized the accumulation of within-task fatigue, allowing us to more directly examine how self-reported fatigue symptoms relate to prospective effort valuation rather than transient fatigue induced by task performance itself. Some previous evaluations of cognitive effort costs intermingled decision-making and exertion, or provided only a limited number of effort levels for comparison (15,23,42). Other studies focused primarily on performance metrics to explain why individuals experiencing depressive episodes exert less effort for equivalent rewards (17). Such approaches may be less sensitive to individual differences in effort valuation, potentially contributing to prior mixed findings regarding differences between individuals with MDD and healthy controls (25, 26, 27). Nonetheless, our design enabled us to identify fatigue as an important factor influencing the valuation of prospective cognitive effort in depression. Although our findings are consistent with altered effort valuation in individuals with greater fatigue-related symptoms, the current analyses do not directly estimate subjective effort cost functions. Future work using formal computational modeling approaches will be important for a more precise characterization of the mechanisms linking fatigue to effort-based decision-making.

A limitation of the present study is that healthy control participants did not undergo a formal structured clinical interview comparable with the DIGS assessment used in the MDD group. Although control participants were screened for self-reported psychiatric and neurologic disorders and exhibited low BDI and MFIS scores, the possibility of unrecognized subclinical symptoms cannot be fully excluded. In addition, this study was not specifically designed or powered to evaluate the effects of medication status on effort-based decision-making or fatigue-related symptom expression. Future studies with larger and more clinically stratified samples will be important for determining how psychotropic medications may influence cognitive effort valuation in MDD.

Previously, work examining effort-cost decision-making in MDD has focused on decreased sensitivity to reward as a central explanation of choice differences between those with depression and their control counterparts (15,18,23). Although the effect of reduced sensitivity to anticipatory and consummatory reward in MDD is well documented (43, 44, 45, 46), we did not find an effect of increased MDD symptom severity or fatigue on reward value in this study. Instead, in the context of our study, we found that increased effort cost was a significant component modulated by individuals’ feelings of fatigue. These findings are consistent with recent studies that have more fully sampled the space of prospective reward and effort value (16,17) and support the need for further studies that incorporate additional factors that may contribute to effort cost, such as effort perception and task difficulty.

Our study suggests that fatigue plays a significant role in decision-making in healthy individuals and those with MDD. Fatigue is a central symptom of MDD (1), and it has been postulated that it may contribute to other documented symptoms, such as cognitive deficits or inability to disengage from negative stimuli (47). However, how fatigue-related feelings in MDD contribute to behavioral differences remains understudied. Here, we present evidence that differences in effort-based decision-making may be exacerbated by increased fatigue, rather than mood symptoms alone.

Although our study shows a link between fatigue symptoms and changes in decision-making, more research is needed to understand how fatigue impacts this process. Because fatigue is common in many neurologic and psychiatric conditions, it is important to determine whether its effects in MDD differ from those in other disorders. In the future, it will be valuable to explore whether the same disruptions that cause fatigue also contribute to other symptoms in MDD, or whether fatigue itself mediates these symptoms. Understanding these dynamics could provide insight into the recently observed transdiagnostic differences in effort-based decision-making among bipolar disorder, schizophrenia, and MDD (25,26,40,48).

### Conclusions

Our study demonstrates that fatigue plays an important role in prospective cognitive effort-based decision-making and that individuals with MDD experience significantly greater levels of fatigue than healthy controls. Across participants, fatigue severity modulated choice behavior, such that higher fatigue was associated with greater sensitivity to prospective cognitive effort costs. These findings suggest that fatigue symptoms may contribute to individual differences in cognitive effort allocation that are not fully explained by diagnostic status or depressive symptom severity alone. More broadly, our results help clarify how fatigue may influence motivated behavior in psychiatric disorders, advancing our understanding of depression and potentially informing future treatment approaches targeting motivational impairments and fatigue-related dysfunction.

## Supplementary Material

Supplemental Figure S1 and Tables S1–S13

Key Resources Table
