# Supplementary material for: Fatigue Symptoms Influence Effort-Based Decision-Making in Major Depressive Disorder
Source: Biol Psychiatry Glob Open Sci. 2026 Jun 24;6(5):100780. doi: 10.1016/j.bpsgos.2026.100780 (PMC13452981; doi:10.1016/j.bpsgos.2026.100780)
Supplement: Supplementary file 1 [file mmc1.pdf]

SUPPLEMENTARY INFORMATION

Fatigue Symptoms Influence Effort-Based Decision-Making in Major Depressive Disorder

Steward *et al.*

Figure S1. Demographic characteristics of the healthy and MDD cohorts. 3

Table S1. Counts of participants on psychotropic medications in the MDD Cohort. No persons in the healthy cohort reported a history of psychotropic medications. 4
Table S2. Paradigm Choice Set. Unique choices presented to participants. Participants would choose whether to perform one of the offers below for the stated effort and reward, or to perform the default 1-back task for a $1 reward. 5
Table S3. No Significant Difference in n-back Performance Between Cohorts. N-Way ANOVA Type III Sum of Squares 6
Table S4. No Significant Difference in n-back Difficulty Perception Between Cohorts. N-Way ANOVA Type III Sum of Squares 6
Table S5. The Effect of Diagnostic Group (Control/MDD) on Choice. The following table provides results for the model: C ~ E*cohort + R*cohort + (1 | subj)... 6
Table S6. Using Task Accuracy as an Analog for Effort in Choice. The following table provides results for the model: C ~ Acc*Cohort + R*Cohort + (1 | subj) 6
Table S7. The Effect of Total MFIS Score on Choice. The following table provides results for the model: C ~ E*MFIS + R*MFIS + (1 | subj) 7
Table S8. The Effect of Total BDI Score on Choice. The following table provides results for the model: C ~ E*BDI + R*BDI + (1 | subj).. 7
Table S9. The Effect of the MFIS Cognitivel Subscale Score on Choice. The following table provides results for the model: C ~ E*MFIS_Cog + R* MFIS_Cog + (1 | subj) 7
Table S10. The Effect of the BDI Somatic Subscale Score on Choice. The following table provides results for the model: C ~ E*BDI_Som + R*BDI_Som + (1 | subj).. 8
Table S11. The Effect of the BDI Cognitive Affective Subscale Score on Choice. The following table provides results for the model: C ~ E*BDI_CogAff + R* BDI_CogAff + (1 | subj) 8
Table S12. The Effect of the MFIS Physical Subscale Score on Choice. The following table provides results for the model: C ~ E*MFIS_Phys + R* MFIS_Phys + (1 | subj) 8
Table S13. The Effect of the MFIS Psychosocial Subscale Score on Choice. The following table provides results for the model: C ~ E*MFIS_PsychoSoc + R* MFIS_PsychoSoc + (1 | subj) 9

Figures

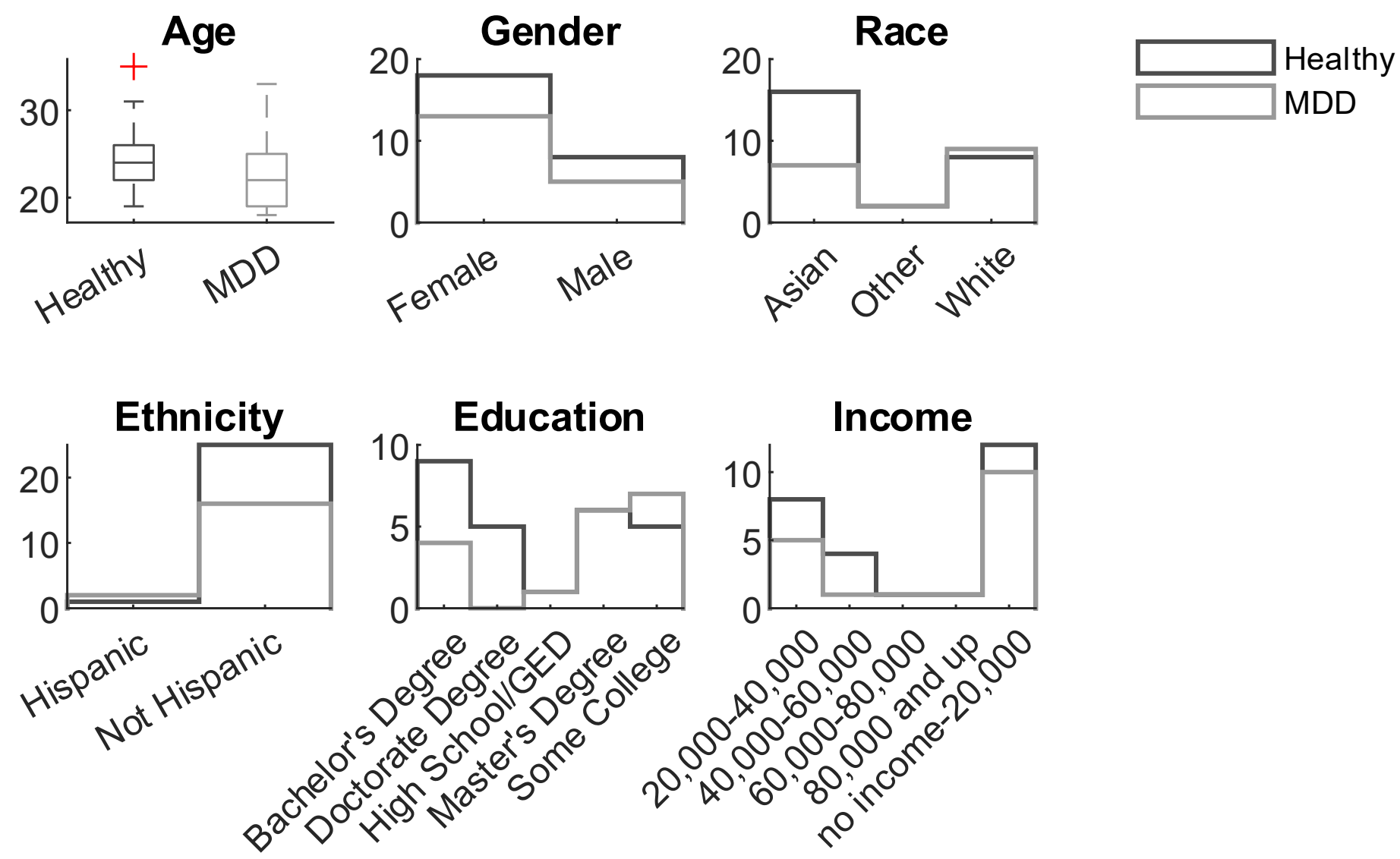

Figure S1. *Demographic characteristics of the healthy and MDD cohorts.*

Tables

Table S1. Counts of participants on psychotropic medications in the MDD Cohort. *No persons in the healthy cohort reported a history of psychotropic medications.*

Medication	***Participant Count***
bupropion	5
buspirone	1
escitalopram	6
gabapentin	1
none	4
paroxetine	1
sertraline	2
venlafaxine	3

Table S2. Paradigm Choice Set. *Unique choices presented to participants. Participants would choose whether to perform one of the offers below for the stated effort and reward, or to perform the default 1-back task for a $1 reward.*

**Effort Level (*n*)**	**Reward ($)**
2	1
3	1
4	1
2	2
3	2
4	2
2	3
3	3
4	3
2	4
3	4
4	4
2	5
3	5
4	5
2	6
3	6
4	6
2	7
3	7
4	7
2	8
3	8
4	8

Table S3. No Significant Difference in n-back Performance Between Cohorts. *N-Way ANOVA Type III Sum of Squares*

	Sum of Squares	DF	Mean Squares	F	P Value
n-back Level	0.683	3	0.228	23.48	3.414 E -12
Cohort	0.001	1	0.001	0.12	0.730
Participant (Nested in Cohort)	0.779	42	0.019	1.91	0.003
Error	1.251	129	0.010		
Total	2.715	175			

Table S4. No Significant Difference in n-back Difficulty Perception Between Cohorts. *N-Way ANOVA Type III Sum of Squares*

	Sum of Squares	DF	Mean Squares	F	P Value
n-back Level	220.16	3	73.388	33.23	5.569 E -16
Cohort	1.97	1	1.968	0.89	0.347
Participant (Nested in Cohort)	65.35	42	1.556	0.70	0.90
Error	284.91	129	2.208		
Total	572.39	175			

Table S5. The Effect of Diagnostic Group (Control/MDD) on Choice. *The following table provides results for the model: C ~ E*cohort + R*cohort + (1 | subj)*

Predictors	Estimate	SE	t Statistic	DF	P Value
E	-4.126	0.373	-11.054	1037	6.27E-27
R	4.870	0.447	10.903	1037	2.80E-26
Control/MDD	-0.399	0.529	-0.755	1037	0.450
E × Control/MDD	-0.758	0.628	-1.207	1037	0.228
R × Control/MDD	0.039	0.735	0.054	1037	0.957

Table S6. Using Task Accuracy as an Analog for Effort in Choice. *The following table provides results for the model: C ~ Acc*Cohort + R*Cohort + (1 | subj)*

Predictors	Estimate	SE	t Statistic	DF	P Value
Accuracy	-0.555	0.386	-1.437	1037	0.151
R	2.948	0.321	9.190	1037	2.09E-19
Control/MDD	-19.024	2.196	-8.662	1037	1.75E-17
Accuracy × Control/MDD	20.144	2.414	8.345	1037	2.25E-16
R × Control/MDD	1.006	0.580	1.733	1037	0.083

Table S7. The Effect of Total MFIS Score on Choice. *The following table provides results for the model: C ~ E*MFIS + R*MFIS + (1 | subj)*

Predictors	Estimate	SE	t Statistic	DF	P Value
E	-3.510	0.519	-6.766	1037	2.21E-11
R	5.532	0.632	8.753	1037	8.29E-18
MFIS	-0.115	0.619	-0.185	1037	0.853
E × MFIS	-2.214	1.011	-2.190	1037	0.029
R × MFIS	-1.283	1.134	-1.131	1037	0.258

Table S8. The Effect of Total BDI Score on Choice. *The following table provides results for the model: C ~ E*BDI + R*BDI + (1 | subj)*

Predictors	Estimate	SE	t Statistic	DF	P Value
E	-4.589	0.454	-10.114	1037	5.34E-23
R	5.161	0.525	9.836	1037	6.91E-22
BDI	-0.650	0.781	-0.833	1037	0.405
E × BDI	0.573	1.022	0.561	1037	0.575
R × BDI	-0.848	1.190	-0.713	1037	0.476

Table S9. The Effect of the MFIS Cognitive Subscale Score on Choice. *The following table provides results for the model: C ~ E*MFIS_Cog + R* MFIS_Cog + (1 | subj)*

Predictors	Estimate	SE	t Statistic	DF	P Value
E	-3.609	0.506	-7.137	1037	1.79E-12
R	5.738	0.613	9.360	1037	4.79E-20
MFIS_Cog	-0.054	0.682	-0.079	1037	0.937
E × MFIS_Cog	-2.324	1.088	-2.136	1037	0.033
R × MFIS_Cog	-1.915	1.188	-1.612	1037	0.107

Table S10. The Effect of the BDI Somatic Subscale Score on Choice. *The following table provides results for the model: C ~ E*BDI_Som + R*BDI_Som + (1 | subj)*

Predictors	Estimate	SE	t Statistic	DF	P Value
E	-4.254	0.468	-9.093	1037	4.79E-19
R	4.778	0.542	8.809	1037	5.24E-18
BDI_Som	-0.896	0.819	-1.094	1037	0.274
E × BDI_Som	-0.437	1.174	-0.373	1037	0.710
R × BDI_Som	0.416	1.372	0.304	1037	0.762

Table S11. The Effect of the BDI Cognitive Affective Subscale Score on Choice. *The following table provides results for the model: C ~ E*BDI_CogAff + R* BDI_CogAff + (1 | subj)*

Predictors	Estimate	SE	t Statistic	DF	P Value
E	-4.625	0.460	-10.064	1037	8.55E-23
R	5.199	0.533	9.762	1037	1.35E-21
BDI_CogAff	-0.648	0.772	-0.840	1037	0.401
E × BDI_CogAff	0.674	1.020	0.661	1037	0.509
R × BDI_CogAff	-0.944	1.191	-0.793	1037	0.428

Table S12. The Effect of the MFIS Physical Subscale Score on Choice. *The following table provides results for the model: C ~ E*MFIS_Phys + R* MFIS_Phys + (1 | subj)*

Predictors	Estimate	SE	t Statistic	DF	P Value
E	-3.535	0.479	-7.381	1037	3.21E-13
R	4.999	0.579	8.627	1037	2.34E-17
MFIS_Phys	-0.224	0.678	-0.331	1037	0.741
E × MFIS_Phys	-2.371	1.050	-2.259	1037	0.024
R × MFIS_Phys	-0.193	1.194	-0.162	1037	0.871

Table S13. The Effect of the MFIS Psychosocial Subscale Score on Choice. *The following table provides results for the model: C ~ E*MFIS_PsychoSoc + R* MFIS_PsychoSoc + (1 | subj)*

Predictors	Estimate	SE	t Statistic	DF	P Value
E	-4.026	0.453	-8.898	1037	2.49E-18
R	5.223	0.531	9.839	1037	6.71E-22
MFIS_PsychoSoc	-0.292	0.747	-0.391	1037	0.696
E × MFIS_PsychoSoc	-1.250	1.057	-1.182	1037	0.237
R × MFIS_PsychoSoc	-0.981	1.175	-0.835	1037	0.404
